# Climate-smart livestock nutrition in semi-arid Southern African agricultural systems

**DOI:** 10.3389/fvets.2025.1507152

**Published:** 2025-02-11

**Authors:** Felix Fushai, Teedzai Chitura, Oyegunle Emmanuel Oke

**Affiliations:** ^1^Department of Animal Science, Faculty of Science, Engineering and Agriculture, University of Venda, Thohoyandou, Limpopo, South Africa; ^2^Department of Animal Physiology, Federal University of Agriculture, Abeokuta, Nigeria

**Keywords:** climate-smart agriculture, climate-smart feeding, climate-smart feedstuffs, circular feed use, nutritional bioenergetics, heat stress

## Abstract

Climate change is disrupting the semi-arid agricultural systems in Southern Africa, where livestock is crucial to food security and livelihoods. This review evaluates the bioenergetic and agroecological scope for climate-adaptive livestock nutrition in the region. An analysis of the literature on climate change implications on livestock nutrition and thermal welfare in the regional agroecological context was conducted. The information gathered was systematically synthesized into tabular summaries of the fundamentals of climate-smart bioenergetics, thermoregulation, livestock heat stress defence mechanisms, the thermo-bioactive feed components, and potentially climate-smart feed resources in the region. The analysis supports the adoption of climate-smart livestock nutrition when conceptualized as precision feeding combined with dietary strategies that enhance thermal resilience in livestock, and the adaptation of production systems to the decline in availability of conventional feedstuffs by incorporating climate-smart alternatives. The keystone potential climate-smart alternative feedstuffs are identified to be the small cereal grains, such as sorghum (*Sorghum bicolor*) and pearl millet (*Pennisetum glaucum*) as dietary energy sources, the native legumes, such as the cowpea (*Vigna unguiculata*) and the marama bean (*Tylosema esculentum*) as protein sources, wild browse Fabaceae trees such as *Vachellia* spp. and *Colophospermum mopane*, which provide dry season and drought supplementary protein, minerals, and antioxidants, the non-fabaceous tree species such as the marula tree (*Sclerocarya birrea*), from which animals consume the energy and electrolyte-rich fresh fruit or processed pulp. Feedstuffs for potential circular feeding systems include the oilseed cakes from the macadamia (*Macadamia integrifolia*) nut, the castor (*Ricinus communis*), and Jatropha (*Jatropha curcas*) beans, which are rich in protein and energy, insect feed protein and energy, primarily the black soldier fly larvae (*Hermetia illucens*), and microbial protein from phototrophic algae (*Spirulina*, *Chlorella*), and yeasts (*Saccharomyces cerevisiae*). Additives for thermo-functionally enhanced diets include synthetic and natural anti-oxidants, phytogenics, biotic agents (prebiotics, probiotics, synbiotics, postbiotics), and electrolytes. The review presents a conceptual framework for climate-smart feeding strategies that enhance system resilience across the livestock-energy-water-food nexus, to inform broader, in-depth research, promote climate-smart farm practices and support governmental policies which are tailored to the agroecology of the region.

## 1 Introduction

Climate-smart agriculture (CSA) promotes agricultural practices which enhance food security and livelihoods while mitigating and adapting agro-systems to the challenges posed by climate change. This broad climate smart approach seeks to build climate resilience into agricultural systems, to ensure they are equipped to withstand extreme climate-related disruptions (1, 2). It integrates climate-adaptation strategies, and mitigation of the causal factors (3, 4). Maluleke and Mokwena (5), Maluleke and Mokwena (6) indicated that semi-arid Southern Africa faces uniquely adverse climate impacts due to its highly climate-dependent livestock systems. This is compounded by extreme temperatures, prolonged droughts, and erratic rainfall which are common in the region (7). These adverse climatic shifts exacerbate the inherent water scarcity, heat stress on livestock, and reduce forage and food crop yields, significantly compromising established precision livestock nutrition, and may offset the genetic progress in the productivity of livestock and food crops (8, 9). The shortage of high-quality feeds in turn increases producer reliance on less efficient alternatives (10). In the animal body, energy efficiency, and inversely, the heat increment of feeding, and the cellular defences against heat-induced oxidative damage are all strongly influenced by feed quality (11). As stated by Sammad et al. (12), understanding the dietary influences on the animal’s thermoregulation, and the impact of heat stress on its health and productivity is crucial to climate-smart feeding. Therefore, addressing climate change impacts on the production of high-quality feedstuffs is crucial to supporting livestock thermal welfare, sustenance of high productivity, reducing greenhouse gas (GHG) emissions, and enhancing systems’ sustainability (13).

Millions of people in Southern Africa depend heavily on livestock production for their livelihoods, supporting jobs, economic stability, and food security. The livestock systems in the area are varied and include commercial operations, pastoral systems, and smallholder mixed crop-livestock systems (14). However, the negative consequences of climate change, characterized by extended droughts, unpredictable rainfall patterns, and rising temperatures, pose a growing threat to these systems. The productivity and resilience of livestock are weakened by these climate stressors, which also increase competition for water resources, decrease pasture productivity, and worsen feed shortages (15). Due to their reliance on massive grazing systems and rain-fed agriculture, Southern Africa’s semi-arid landscapes are especially susceptible to these effects. For instance, extended droughts in nations such as Zimbabwe, Namibia, and Botswana have resulted in a sharp decline in the productivity of the rangelands, making different ruminant feeding techniques necessary to sustain the livestock. In smallholder systems, the lack of reasonably priced, high-quality feeds during dry seasons frequently leads to decreased output and poor animal health, which fuels poverty cycles.

These difficulties highlight how urgently climate-smart feeding methods adapted to Southern African environments are needed. To address these questions, this review introduces the concept of climate-smart livestock nutrition (CSLN). This involves the selection of feedstuffs based on both nutrient content and thermo-functional properties, and the climate change implications of their production and utilization, to formulate diets that promote thermal regulation, reduce oxidative stress, and minimize GHG emissions, to ensure viable and sustainable feed resources. Climate-smart livestock feeding incorporates novel techniques and feed materials to improve sustainability, resilience, and production (16). Utilizing locally accessible feed resources, such as crop wastes, agro-industrial byproducts, drought-tolerant forage species, and cutting-edge feed technologies such as insect-based proteins and biofortified feedstuffs are possible options to enhance CSLN through climate mitigation by circular feed utilization (17, 18).

To succeed, CSLN requires evidence-driven policies to support research, promote best practices, including the enforcement of regulations on livestock-linked GHG emissions, land, water, and energy use (1). This review explores the biophysical basis and scope for CSLN and outlines a conceptual framework for its implementation in semi-arid Southern Africa, to guide research, farm practices, and policy development.

## 2 Livestock production, climate change, and climate-smart agriculture

The Earth’s atmosphere is primarily composed of nitrogen (78%), oxygen (21%), and trace (1%) quantities of other gases, including argon, carbon dioxide (CO_2_), methane (CH_4_), and water vapour (19). The phenomenon of rising global average temperatures, or global warming is attributed to the green-house effect of water vapour, carbon dioxide, methane, nitrous oxide and ozone, the greenhouse gases (GHG) which trap heat from the sun, leading to an increase in the earth’s surface temperature, to influence the earth’s weather patterns (rainfall, temperature droughts), a phenomenon described as climate change (20). Climate-smart agriculture is a concept that is contiguous to the older notion of sustainable agriculture, which emerged in the context of adverse climate change. It provides an over-arching conceptual framework for transforming contemporary agriculture to sustain food security despite climate change (2). The FAO (1) defined CSA as multifaced, to encompass a sustainable increase in agricultural productivity, reduction of climate change vulnerability (adaptation) and GHG emissions (mitigation), while enhancing food security and livelihoods.

Livestock production plays an important role in providing livelihoods and supporting economies at the household, local, national and global scales. Therefore, the sector sits at the center of climate-smart agriculture, for food security (21). The debate on the contribution of livestock production to climate change remains controversial (22). Estimates suggest a contribution of as much as 18% of the GHG emissions into the earth’s atmosphere, mostly through enteric fermentation, manure degradation and feed production (10). The GHG emissions depend on the livestock species, systems and practices in relation to inefficiencies, and intensification. Thornton and Herrero (10) reported that approximately one third of the emissions are agronomic (land use and feed production), one-third from manure (nitrous oxide, methane) management, a quarter from enteric methane, and the rest from other livestock-related functions. Herrero et al. (23) estimated that two-thirds of the global emissions come from ruminant systems in the developing world. There is scant data on African livestock systems. However, in sub-Saharan Africa, Graham et al. (24) indicated that typically high emissions per unit product are attributed to low animal productivity, poor animal health, and low-quality feeds. This scenario presents scope to mitigate climate change through efficient feed production and feeding, instead of scaling down production and consumption of the much-needed animal products.

## 3 Agroecological scope of semi-arid Southern Africa

Climate-Smart Livestock Nutrition (CSLN) is novel in its emphasis on the role of livestock nutrition in mitigating adverse climate change impacts on livestock and the production systems. It seeks to enhance thermal tolerance in livestock, and to reduce greenhouse gas emissions (GHG) from enteric or rumen fermentation, from upstream inputs into feed production such as such fertilizers and irrigation, and from downstream (manure decomposition) emissions. The climate-smart livestock production guidelines of the Food and Agricultural Organization (25) effectively support three primary objectives to anchor the CSLN approach:

- *Adapting to declining feed availability and quality*: Identification of climate resilient crops and varieties, and efficient utilization of feed resources, to address the increasing scarcity and cost of conventional feeds, while maintaining dietary quality.
- *Enhancing livestock resilience to heat stress*: Utilization of dietary ingredients which contain natural mitigants to thermal stress such as antioxidants and electrolytes, to augment body mechanisms for coping with extreme temperatures, and reduce oxidative stress. Additional nutritional interventions include, for example, changing the roughage-concentrate ratio (ruminants) and supplementary dietary fats to ensure adequate energy intake by all livestock despite heat stress.
- *Minimizing the environmental impact:* Develop sustainable feed production, processing and utilization technologies, and increase reliance on circular feed systems, to reduce the ecological footprint of livestock production.

To achieve its objectives in the semi-arid Southern African ecosystems, CSLN demands agro-ecological zone-specific strategies which are tailored to the unique challenges. The agro-ecology is characterized by low and highly variable rainfall (300–600 mm annually), and a mix of soil and vegetation conditions which impose significant constraints to productive, sustainable agriculture. The region increasingly experiences extended dry seasons, with the rain seasons and unpredictable rain, which exacerbates water stress, with high temperatures exceeding 40°C, which further intensify evapotranspiration (26, 27). Climate models project that temperatures will rise faster than the global average, particularly in the low-altitude semi-arid to arid zones, with mean surface warming surpassing global trends in all seasons (28–30). Regions such as the northwest South Africa, Botswana, and Namibia are particularly vulnerable to this accelerated warming (31).

Smallholder farmers in these areas rely on mixed crop-livestock systems, where sheep, goats, and cattle play critical roles in cultural, economic, and food security needs. However, natural rangelands and supplementary crop byproducts, the primary feed resources, are often inadequate during prolonged dry periods. To address these challenges, innovative strategies such as water-saving techniques, forage diversification, and drought-resilient crops are increasingly adopted, which provide potential climate-smart animal nutrition solutions to these harsh conditions.

Livestock systems in semi-arid Southern Africa are particularly vulnerable to the disruptive effects of climate change, particularly rising temperatures, prolonged droughts, and erratic rainfall patterns (5, 7). These changes exacerbate water scarcity, heat stress, and declines in forage and crop yields, negatively impacting livestock nutrition and thermal welfare. The retrogressive effects on precision livestock nutrition compel producers to turn to unconventional feed resources, often inefficient, to sustain or intensify production. This practice, however, risks increasing livestock-generated greenhouse gas (GHG) emissions (32), which demands urgency in adopting strategic interventions such as those advocated by CSLN.

Heat stress poses a particularly significant challenge, with ambient temperatures frequently exceeding the thermal comfort zones of livestock species. For instance, poultry experience stress above 26°C, while cattle are affected when temperatures rise beyond 25–30°C (33–35). Heat stress reduces feed intake, metabolic efficiency, and overall productivity, resulting in financial losses and animal welfare concerns. The CSLN approach addresses these issues by incorporating heat-mitigating strategies, such as diets enriched with antioxidants, electrolytes, and essential nutrients, to enhance livestock thermotolerance.

Beyond thermal resilience, CSLN contributes to broader climate adaptation by ensuring livestock remain productive in sustainable systems despite extreme climatic conditions to support food security and economic stability in vulnerable regions. Effective promotion of CSLN practices requires targeted investments, including funding for the development of heat-mitigating feed additives, integrating them into smallholder and commercial feeding systems, and providing financial incentives to encourage adoption. Additionally, robust support for farmer training programs and extension services is crucial to scaling up CSLN interventions and achieving widespread impact.

## 4 Conceptual framework for implementing climate-smart livestock nutrition in Southern Africa

A framework for the implementation of effective CSLN should integrate the underpinning principles of nutritional bioenergetics, thermoregulation, oxidative stress, and sustainable feed systems to address the three core objectives: adapting to declining feed availability, enhancing livestock resilience to heat stress, and minimizing environmental impact. A possible conceptual framework is outlined in Table 1.

**Table 1:** A conceptual framework for climate-smart livestock nutrition interventions in Southern Africa.

Principles	Concepts	Mechanistic pathways	Impact	Climate-smart interventions	Indicators	References
Bioenergetics	Energy efficiency & heat production	Dietary inefficiencies increase heat production, intensifying heat stress and reducing productivity.	Heat stress from inefficient energy use increases the environmental heat load to limit performance,	Formulate energy-efficient diets with low-heat increment feedstuffs, feedings in cooler parts of the day.	Serum glucose, lactate, rectal temperature.	(10, 13)
Metabolic & hormonal acclimation	Endocrine balance during heat stress	Heat stress alters hormone levels, reducing feed intake and energy balance.	Imbalance in energy availability reduces growth and reproduction.	Manage adverse responses though dietary modifications	Insulin, cortisol, T3, and T4 levels.	(8, 12)
Thermoregulation	Neuroendocrine signaling	Heat stress disrupts neuroendocrine signals affecting metabolism and behavior.	Altered signaling reduces feed intake and changes metabolic responses.	Manage adverse responses though dietary modifications	Dopamine, norepinephrine, cortisol levels.	(7, 10)
Oxidative stress	Reactive oxygen species (ROS) control	Heat stress elevates ROS levels, causing cellular oxidative damage.	Oxidative damage decreases immune responses and overall productivity.	Supplement diets with antioxidants (vitamin E, curcumin) to neutralize ROS and reduce oxidative stress.	ROS levels.	(9, 13)
Heat shock response	Heat shock proteins (HSPs)	HSPs protect cells from heat-induced damage by stabilizing protein structure.	Reduced HSPs increase susceptibility to cellular damage and stress.	Nutritional approaches to support HSP regulation using supplements to enhance resilience to heat stress.	HSP70 levels, HSF-1 activity.	(11, 18)
Epigenetics	Epigenetic modifications for thermal tolerance	Heat triggers DNA methylation and histone protein modifications to influence gene expression.	Epigenetic alterations can either enhance or reduce stress resilience.	Use epigenetic markers in selective breeding for thermal tolerance; methyl donors such as choline, folate, betaine in diets to modulate gene expression.	DNA methylation patterns, histone acetylation levels.	(1, 10)
Nutrigenomics	Nutrient effects on gene expression	Nutrients regulate genes which control stress tolerance, metabolism, and immunity.	Improves feed efficiency, heat resilience, and immune function.	Supplement with selenium, omega-3 fatty acids, and antioxidants to modulate stress-related gene expression.	Gene expression levels (HSPB8, SERPINH1), antioxidant enzyme activity.	(7, 8)
Nutrigenetics	Genetic variation & dietary responses	Genetic polymorphisms affect nutrient metabolism and thermal tolerance	Inefficient nutrient absorption or metabolism due to genetic variation can reduce performance in heat stress.	Tailor diets to match genetic profiles (precision feeding) to improve nutrient efficiency and stress resilience.	Nutrigenetic markers for nutrient metabolism and heat resilience.	(10, 12)
Sustainable feeds	Climate-smart feedstuffs	Climate change reduces conventional feed availability, increasing the use of alternative, sustainable feed sources.	Decreased feed availability leads to reduced productivity and higher methane emissions.	Use insect feed, climate-resilient forage crops, and alternative protein sources to lower greenhouse gas (GHG) emissions and improve resilience.	Feed quality (protein, fiber content), methane emissions.	(1, 17)
Antioxidant defence	Exogenous antioxidants	Bioactive dietary compounds reduce oxidative stress and enhance cellular protection.	Elevated oxidative stress reduces animal health, productivity, and immune function.	Use of supplements to enhance antioxidant defences.	Plasma antioxidant levels, oxidative stress biomarkers.	(9, 13)
Water-electrolyte balance	Hydration & electrolyte homeostasis	Heat stress accelerates fluid loss and causes electrolyte imbalances	Dehydration and imbalances lead to reduced productivity and health.	Supplement electrolytes (Na, K, Cl) to maintain hydration and heat tolerance.	Plasma osmolality, Na+, K+, Cl − levels, feed intake, body weight and feed efficiency	(3, 12)

### 4.1 Bioenergetics of climate-smart livestock nutrition

Effective climate-smart livestock nutrition solutions require adequate understanding and application of the fundamental bioenergetics. Depending on the species, the thermal homeostasis and nutritional bioenergetics of livestock are profoundly influenced by dietary factors. Diet influences the animal’s thermal regulation, including modulation of heat stress as well as defence mechanisms at the molecular, cellular and higher-levels (36). Bioenergetics is therefore, fundamental to the optimum dietary management of the thermal welfare of livestock (37). An array of complex dietary factors is known to influence energy extraction from feeds through both the quantum, and profile of energy substrates, and the intermediates, as they flow into central metabolism, to determine the overall dietary energy efficiency, and inversely, heat production (38). These bioenergetic interactions are summarized in Table 2. Therefore, climate-smart feed characterization and diet formulation should prioritize the influences on energy consumption and efficiency, and the associated heat production, in relation to heat stress, and its effect on the animal’s biochemical and physiological functions which determine productivity. In semi-arid Southern Africa, livestock are frequently subjected to environmental stresses such as heat and feed scarcity, factors which can drastically change their energy needs and utilization (15). For example, increased thermoregulation due to heat stress can increase maintenance energy demands, reducing the energy available for growth, reproduction, or milk production.

**Table 2:** Bioenergetics, heat stress, and thermoregulation in livestock.

Bioenergetic factors	Thermal responses and mechanisms	References
Thermal stress	*Climate-induced heat stress*: Increased frequency of extreme temperatures in prolonged heatwaves negatively affects livestock welfare and productivity.	(39, 40)
	*Oxidative stress*: Heat stress enhances reactive oxygen species (ROS) production, leading to cellular lipid peroxidation and protein denaturation, which impair growth; mitochondria are primary ROS sources.	(61, 63)
	*Altered energy metabolism*: Elevated maintenance energy demands coupled with decreased feed intake and nutrient absorption compromise growth under heat stress.	(40)
Neuro-endocrine regulation	*Neuroendocrine responses*: Dopamine and norepinephrine modulate stress responses via the hypothalamic–pituitary–adrenal (HPA) axis and sympathetic-adrenal-medullary (SAM) systems.	(45)
	*Endocrine responses*: Reduced levels of T3 and T4 hormones lower heat production; increased insulin promotes heat shock protein (HSP) expression, enhancing stress adaptation.	(39, 42)
Molecular defences	*Heat Shock Proteins (HSPs)*: HSPs act as molecular chaperones to facilitate proper protein folding and prevent aggregation during thermal stress; HSF-1 regulates HSP expression and serves as a biomarker for resilience.	(67)
	Antioxidant defense systems:	(78)
	*Enzymatic*: Key enzymes (SOD, catalase, glutathione peroxidase) convert ROS into less harmful molecules, protecting cells from oxidative damage.	
	*Non-Enzymatic*: Plant-derived and synthetic bioactive dietary compounds such as glutathione, vitamin E, polyphenols, and flavonoids neutralize ROS and support cellular repair, mitigating oxidative stress.	(74, 76, 87, 205)

### 4.2 Metabolic and hormonal acclimation to a hot environment

Reduced feed intake is the direct mechanism through which heat stress affects production and reproduction, coupled with altered endocrine status, increased maintenance requirements, decreased rumination and or nutrient absorption (39). These mechanisms contribute to a net decrease in nutrient/energy assimilation. For example, lactating heifers lose body weight during periods of extreme heat stress, which is at least partially explained by a drop in energy intake, coupled with an increase in energy expenditure for maintenance (40).

Hormones are linked to the body’s acclimatory reaction to heat stress (39). These include growth hormone, prolactin, thyroid hormones, glucocorticoids, and mineralocorticoids. The thyroid hormones, T4 and T3, have drawn the most interest because they constitute a key acclimatization mechanism. Mammals that have evolved to warmer temperatures are known to follow the pattern of decreased endogenous thyroid hormone levels during heat acclimation as a means of reducing endogenous heat generation (41). Research shows that insulin is also involved in acclimation with animals under heat stress showing greater insulin levels, even if they consume less feed. The function of insulin in inducing heat shock proteins may partially explain this conundrum (42). For example, HSP70 expression is positively correlated to circulating insulin levels (43), and adaptation to hypoxia requires both HSP90 and insulin responses (44).

The neurotransmitters dopamine and norepinephrine are involved in modulating thermoregulation during heat stress (45). They influence the physiological and behavioral responses to thermal stress, which makes them relevant to the overall stress management in livestock. Understanding neuroendocrine pathways is therefore essential for developing effective climate-smart feeding strategies. By targeting these pathways, it is possible to optimize feeding practices to enhance animals’ resilience to heat stress (34). The central nervous system plays a major role in hormone regulation. The hypothalamic–pituitary-thyroid axis, the sympathetic-adreno-medullary axis, the hypothalamic–pituitary–adrenal axis (HPA), and the hypothalamic–pituitary-gonadal axis can all be affected by heat stress. The primary neurosecretory systems triggered by stress include the HPA and sympathetic-adrenal-medullary (SAM) system, among others (46, 47). As reported by Beede and Collier (48), when animals are thermally challenged, the endocrine system, a vital component in the coordination of metabolism, undergoes significant modifications. Collier et al. (39) stated that the hypothalamic–pituitary–adrenal axis represents a crucial element of the body’s acclimatory reaction to heat stress while the thyroid hormones, specifically T3 and T4, are essential for animals’ proper growth, differentiation, and metabolism. They are essential for controlling body temperature, energy intake, and thermal metabolic adaptability (49). Elevated ambient temperature dramatically diminishes T3 secretion while augmenting T4 synthesis in chickens (50). Yousef and Johnson (51) identified a synergistic impact between the decrease in thyroid hormones and the decreased level of growth hormone in plasma which aids the body’s urge to minimize heat production. Thyroid atrophy and diminished secretory function, and other thyroid-related conditions may be direct impacts of heat stress (52).

One of the primary hormones involved in the stress response is cortisol which primarily supports gluconeogenesis by promoting protein metabolism (53), which turns proteins into amino acids. Sejian and Srivastava (54) pointed out that in the liver, muscle, and adipose tissue, the cortisol produced by the adrenal cortex promotes the breakdown and release of glucose, amino acids, and fat. Almost every biological function that is impacted by stress is regulated by cortisol, including behavior, metabolism, immunological response, and reproduction. The goal of these hormonal reactions is to increase the capacity to withstand stress. Elevated blood cortisol levels due to high temperatures have been reported to slow down the rate at which heat is produced metabolically (54, 55).

Somatostatin is stimulated by corticotropin-releasing hormone, which may be a major mechanism explaining heat-stressed animals’ decreased thyroid and growth hormone levels (56). Glucocorticoids in dairy cattle fall during acclimatization at high temperatures and were lower in animals that had been thermally acclimated than in controls (57, 58). Heat stress stimulates the hypothalamic axis, which reduces animal feed intake by upregulating the production of adiponectin and leptin as well as the expression of their receptors (59). The receptor and expression of the Neuritin B gene could be enhanced by the thermal challenge (60).

### 4.3 Oxidative stress and cell damage

A biological system constantly produces free radicals, some of which are necessary for physiological functions. The mitochondria are the primary location of aerobic cellular reactive oxygen species (ROS) generation and use more than 90% of the cellular oxygen in undisturbed cells (61, 62). Enzymatic oxidase reactions and the endoplasmic reticulum’s microsomal systems produce ROS (63). When the body produces excessive ROS, lipid peroxidation occurs, which negatively impacts organelles and cell membranes. Superoxide anions, hydrogen peroxide, and hydroxyl radicals are examples of reactive oxygen species that are produced in the mitochondria and function as signaling intermediaries (64).

#### 4.3.1 Heat shock proteins (HSPs)

Heat shock proteins (HSPs) are molecular chaperones which protect cells from heat-induced damage by aiding in protein folding and preventing aggregation (65). These proteins are upregulated in response to various forms of stress, including heat stress, and play a critical role in cellular protection and recovery (66). Heat Shock Factor-1 (HSF-1) is a key regulator of the heat shock response. It orchestrates the transcription of heat shock proteins by binding to heat shock elements (HSEs) in the DNA, thus initiating the cellular stress response (67). In climate-smart feeding, monitoring HSP levels and analyzing HSF-1 activity or its binding to HSEs can serve as indicators of an animal’s capacity to activate protective mechanisms against thermal stress (68).

#### 4.3.2 Antioxidant enzyme defence systems

Under normal conditions, antioxidant enzymes such as catalase, glutathione peroxidases, peroxiredoxins, and superoxide dismutases constantly remove produced ROS (64). When reactive oxygen species are overproduced under stressful situations, hydrogen peroxide is released, creating oxidative stress. This can overload the antioxidant defense system and lead to an imbalance in the redox system (62, 69–71). In addition to increasing plasma corticosterone levels in chickens, stress activates the hypothalamic pituitary adrenal axis (72).

Exogenous and endogenous antioxidants in biological antioxidant defense systems are divided into enzymatic and non-enzymatic categories, which include ROS/RNS scavengers, transition metal chelators, oxidative enzyme inhibitors, and antioxidant enzyme cofactors (73). Low molecular weight antioxidants and antioxidant enzymes are the two main categories of antioxidants. Glutathione peroxidase, catalase and superoxide dismutases and other enzymes are among the most significant antioxidants. Glutathione, flavonoids, carotenoids, vitamin E, vitamin C and other antioxidants are among the most significant low molecular weight antioxidants. These two primary antioxidant systems are crucial in preserving the equilibrium between antioxidant and pro-oxidant agents while reducing oxidative stress (74). Antioxidants work by directly scavenging oxidizing radicals and allowing organisms to repair their damaged biomolecules. Under extreme stress, their activities are restricted (71, 75, 76). A class of proteins called antioxidant enzymes, also known as metalloproteins, catalyze the conversion of reactive oxygen species (ROS) and/or their metabolites into more stable, generally less dangerous species. Antioxidant enzymes are a crucial defensive mechanism against oxidative stress caused by reactive oxygen species (ROS), which damages cell components (77).

While non-enzymatic antioxidants include peroxide decomposers, oxidative enzyme inhibitors, metal chelators, singlet oxygen quenchers, and/or ultraviolet radiation absorbers and enzymatic antioxidants play a protective role by breaking chains of free radicals and scavenging them (73). Superoxide dismutase was the first line of defense against free radicals and maintained cellular redox equilibrium among the potential reactive oxygen species scavengers (78). The SOD is therefore essential for the early stages of defense against ROS-mediated oxidative damage. By facilitating the transformation of superoxide into oxygen and hydrogen peroxide, SOD is an essential component of the defense against free radicals.

Because aerobic organisms produce this enzyme broadly, it is an essential part of the first line of defense against oxidative stress (78). There are three different isoforms of SOD: extracellular SOD3, mitochondrial SOD2, and cytoplasmic SOD1. Most eukaryotic cells have SOD2 and SOD3, with SOD3 being the main isoform identified in the cardiovascular system (79). Nuclear genes encode manganese superoxide dismutase, or SOD2, an antioxidant enzyme. Mutations or disruptions in SOD2 function have been linked to changes in the structure of the mitochondria seen in diseases such as heart failure (80). Reduced SOD2 levels cause ROS to build up and then excessive 4-hydroxynonenal synthesis in the mitochondria (64).

#### 4.3.3 Superoxide dismutase activity and gene expression

Seasonally appropriate feeding, providing feeds high in fiber and fats, supplementing with vitamins and minerals, and offering cold drinking water are some of the dietary changes that promote activities of superoxide dismutase enzyme (68). The orange-yellow lipophilic polyphenolic compound curcumin is extracted from the rhizome of herbs. Its antioxidant and anti-inflammatory qualities have led to its recognition for its important role in the treatment and prevention of cardiovascular diseases (81). Through a variety of methods, the bioactive substance curcumin protects the cardiovascular system from OS. In order to attenuate OS, lower ROS levels, and restore cardiac SOD levels, two important processes implicated are the activation of the PI3K-Akt survival pathway and the SIRT1-FoxO1 pathway (82). Owing mainly to its antioxidant qualities, salvianolic acid, a naturally occurring polyphenolic molecule obtained from *Salvia miltiorrhiza*, demonstrated noteworthy preventive actions against cardiovascular diseases. Salvianolic acid has been shown in numerous studies to be able to postpone the onset of ischemia in animal models of MI by increasing angiogenesis, decreasing infarct size, and enhancing post-infarction contractile performance (83).

In order to coordinate cellular and whole-animal metabolism, gene networks both inside and across cells and tissues react to external heat loads exceeding the thermoneutral zone by sending out intra-and extracellular signals. In Vrindavani cattle (*B. indicus* X *B. taurus*), heat stress response genes (SERPINH1, DNAJ4, FKB4, HSPB8 and HSPH1) were up-regulated at a greater fold change (244). High ambient temperature modulates heat shock protein genes to shield the cells and proteins from a changed metabolism. Induced heat stress causes such changes in physiologic parameters that modify the neuroendocrine system (84). Elayadeth-Meethal et al. (85) investigated the differential expression and molecular mechanism of the HSPA1A gene in dwarf Vechur cattle, Kasaragod cattle, and crossbred cattle in an experimental field context. They concluded that HSPA1A is a possible candidate gene for heat tolerance. The potential for improving thermotolerance through manipulation of the genes regulating HSF1 expression and evaporative heat loss in cattle is suggested by the variation in evaporative heat loss among animals and the crucial role that (heat shock transcription factor 1) HSF1 plays in coordinating thermal tolerance (86).

#### 4.3.4 Exogenous, non-enzymatic antioxidants

Exogenous antioxidants are abundantly found in natural plants and primarily consist of polyphenols and natural flavonoids. Supplementation with exogenous antioxidants exerts potent antioxidant effects by engaging various signaling pathways. These pathways include augmenting the antioxidant capacity of endogenous antioxidant systems, thereby reducing OS, inhibiting ROS production, consequently restraining OS, and activating antioxidant signaling pathways that counteract OS (87).

## 5 Functional compounds in climate-smart feedstuffs

The functional compounds in feeds are the bioactive molecules that have specific physiological or health effects beyond nutrition (88). In the context of thermal stress and oxidative cell damage, there are compounds that help animals cope with heat stress, in support of the internal antioxidant defences, immune responses, and overall biochemical and physiologic resilience to heat stress (245). Climate-smart feeds may contain an array of functional compounds. The natural functional compounds which may be present in climate-smart feedstuffs are indicated in Table 3. To operationalize these solutions to enhance livestock’s health, productivity, and resilience in the face of climate stress, feeding plans need to be modified to fit the resource limitations and production systems of semi-arid Southern Africa. Examples of specific or targeted interventions include the following;

- Antioxidant supplementation: Supplementing with natural antioxidant-rich feedstuffs, such as sorghum bran and sunflower meal, can help reduce the oxidative stress brought on by exposure to heat. These dietary components can be added to concentrates designed to meet animal demands or utilized in silage.
- Phytogenic supplements: To improve gut health and lower oxidative stress, livestock diets can include locally accessible plants with multifunctional bioactive components as feed additions. The phytogenic additives can be combined with crop residues or tree fodder after being processed into meal or extract form.
- Probiotics and prebiotics: Smallholder farmers can use fermented feed technology to add healthy microorganisms to livestock diets, to enhance gut health and nutrient absorption. Fermenting waste grains or grain crop byproducts with supplementary molasses can be an inexpensive way to distribute probiotics.
- Integrated feeding systems: In smallholder systems, functional compounds can be incorporated into domestic feed formulations by employing locally accessible resources. For example, cattle could be supported during the dry season by combining crop residues enriched with neem leaf powder and treated with antioxidants. To target high-value production systems such as dairy or poultry farms, feed manufacturers could create pre-mixed functional ingredient supplements. To cut farmers’ expenses, these might be provided via cooperatives.
- Policy and capacity building: Funding for farmer education programs on processing methods and the advantages of functional compounds is necessary for successful implementation. The feed industry and extension agencies must work together to guarantee that these compounds are affordable and available for a variety of farming methods.

**Table 3:** Dietary chemical defences against thermal stress and oxidative cell damage.

Functional Compound	Examples	Role and application	References
Antioxidants	Vitamin E Selenium Polyphenols Flavonoids	Neutralize free radicals generated during oxidative stress, protecting cells from damage. Can be naturally present in feeds, or as synthetic supplements.	(74, 76)
Electrolytes	Sodium Potassium Magnesium Chloride Bicarbonate Trace minerals	Maintain fluid balance and prevent dehydration under heat stress. Regulate the acid–base balance of the internal environment. Which can be disrupted during thermal stress, ensuring proper muscle and nerve function. Supplementing electrolytes in feed or water helps animals maintain homeostasis during periods of high heat.	(39, 233)
Fatty acids	Omega-3 fatty acids	Anti-inflammatory properties that reduce cellular damage and inflammation caused by oxidative stress during heat exposure. Modulate the immune response and improve overall health and productivity under thermal stress.	(234)
Amino acids	Methionine, Taurine Glutamine	Higher requirement for essential amino acids, primarily methionine, to compensate for the increased protein synthesis during cellular repair after heat related oxidative damage. Precursors for antioxidant molecules (e.g., glutathione) that protect cells from oxidative damage caused by heat stress. Taurine is particularly important in maintaining cellular integrity and hydration under stress.	(42)
Carotenoids	Beta-carotene Astaxanthin Lutein	Beta-carotene helps in removing ROS, protecting cells from oxidative damage. Astaxanthin, found in microalgae, is a potent natural antioxidant, protecting cell membranes from peroxidation under heat stress. Lutein supports eye and skin health, to improve thermal resilience.	(61)
Vitamins	Vitamin C Vitamin A	Vitamin C is a potent antioxidant that removes ROS in tissues exposed to high temperatures. Vitamin A supports the immune system and protects against oxidative damage in epithelial tissues, improving overall animal health under stress conditions.	(76)
Catalytic minerals	Zinc, Copper, Manganese	Trace minerals – co-factors for enzymes involved in antioxidant defense systems. Zinc is a co-factor for superoxide dismutase. Manganese supports mitochondrial function, reducing oxidative damage in cells.	(78, 233)

## 6 Climate-smart feed resources in semi-arid Southern African ecosystems

In the semi-arid regions of Southern Africa, developing sustainable, climate-smart livestock feeding systems is imperative to mitigate severe environmental constraints such as drought, heat stress, and low soil fertility. Climate-smart feedstuffs are those which are cultivated or exploited from the wild, which meet the climate-smart definition, they comply with the agroecological principles of sustainability, productivity and nutritional quality, with least energy, chemicals, and water input (246). The cultivation or wild exploitation of such feed resources should minimize upstream (irrigation, pesticides, fertilizer) greenhouse gasses (mitigation), and promote the most productive, ecologically adapted, climate resilient or drought-tolerant species and varieties (adaptation) (247). Though research on climate-smart feed resources is still limited and fragmented, several promising candidates are emerging, which can be produced or exploited on a large-scale for viable value chains. Strategic incorporation of these feeds, ranging from the drought-tolerant native grain cereals and legumes to biofuel or pharmaceutical oilseed cakes, the wild or cultivated browse trees and the fruit byproducts, and single cell (microbial) protein presents ample opportunity for CSLN (89). A selection of the feed resources which have attracted research attention for potential integration into climate-smart livestock nutrition in semi-arid Southern Africa are profiled in Table 4. The challenges in research are to expand the existing matrix of (conventional) feedstuffs by identifying, and characterizing (nutrients, bioactive compounds) climate-smart alternatives, to facilitate least cost, climate-smart formulation of diets for different livestock (90).

**Table 4:** Profile of representative potential climate-smart in semi-arid Southern African.

Feed category	Keystone candidate climate-smart feedstuffs	Climate-smart attributes	Bioactive compounds	Limitations	Recommended processing	Sources
Small cereal grains	Sorghum (*Sorghum bicolor*), grain	Drought-tolerant, comparatively high yield potential, suitable for low-input agriculture; high energy	Flavonoids, phenolic acids	Low lysine, tannins	Soaking, sprouting, cooking, fermentation.	(91, 235)
	Pearl Millet (*Pennisetum glaucum*), grain	Adapted to dry conditions, high energy, minerals	Phenolic acids	Phytates	Phytase	(93)
Cultivated grain legumes	Cowpea (*Vigna unguiculata*), grain	Tolerant to drought, high protein, enhances soil fertility	Flavonoids, phenolic compounds	Trypsin inhibitors, lectins and other antinutrients	Cooking, soaking, sprouting	(94–96)
Wild grain legumes	Marama Bean (*Tylosema esculentum*), grain	Adapted to arid environments, high protein, high oil (energy) content	Phenolic acids, phytosterols, flavonoids	Trypsin inhibitors and other antinutrients	Heat treatment	(97)
Roots and tubers	Cassava (*Manihot esculenta*) roots	Drought tolerant, high energy	Cyanogenic glycosides	Cyanogenic glycosides (leaves, peels of raw tubers)	Peeling, sun drying, fermentation, cooking	(99, 100)
Wild trees	*Vachelia* spp., pods, twigs, leaves	Drought-resistant, legume, dry season and drought feed, protein-rich, minerals	Polyphenols, flavonoids, terpenoids, glucosinolates, carotenoids	Tannins	Soaking, chemical treatments (lime, sodium bicarbonate)	(101, 102)
	*Colophospermum mopane*, pods, young twigs, leaves	Drought-tolerant, dry season and drought feed, legume, protein-rich, minerals	Polyphenols	Tannins	Soaking, chemical treatments (lime, polyethylene glycol, fermentation)	(101, 102)
	Marula (*Sclerocarya birrea afra*), fruit pulp	Drought-resistant, high in carbohydrates, vitamins, and antioxidants	Vitamin C, phenolic compounds tocopherols (Vitamin E)		Drying, fermentation	(236)
Food nut oilseed cakes	Macadamia (*Macadamia integrifolia*), seed oil cake	High protein, essential fatty acids, high energy	Monounsaturated fats, antioxidants (vitamin E, polyphenols)	High fiber for mnogastrics		(109, 110)
Pharmaceutical oilseed cakes	Castor Bean (*Ricinus communis*) seed oil cake	Drought-tolerant, high protein, essential fatty acids, high energy		Ricin	Heat treatment, fermentation	(111)
	Prickly Pear (*Opuntia ficus-indica*), seed oil cake	Drought-tolerant, protein and energy rich	Antioxidants (betalain pigments, vitamin C), electrolytes	Oxalates	Drying, soaking, water leaching, chemical treatment (lime)	(237, 238)
Biofuel oilseed cakes	Jatropha (*Jatropha curcas*) seed oil cake	Drought-tolerant, high protein content, essential fatty acids		Phorbol esters	Fermentation, heat treatment	(112)
Forages – grass	Napier Grass (*Pennisetum purpureum*)	High yielding, drought-tolerant, suitable for various soil types			Drying, fermentation	(239)
Forages-xerophytes	Spineless Prickly Pear (*Opuntia ficus-indica*) cladodes & waste fruit	Drought-tolerant, high-water content, vitamins, minerals	Antioxidants (betalain pigments), electrolytes	Oxalates	Drying, soaking, water leaching, chemical treatment (lime)	(240)
Insect feed	Black Soldier Fly Larvae (*Hermetia illucens*)	High protein, high energy (full-fat), waste management	Essential amino acids, fat (energy), antimicrobial peptides		Drying, defatting	(120–122)
Microbial derived feeds	Phototrophic algae (e.g., *Spirulina, Chlorella*)	High protein source, rich in carotenoids and polyphenols, omega-3 fatty acids	Carotenoids, polyphenols, DHA, EPA vitamins (B12)	High production cost, scalability challenge, Allergenicity	Drying	(124–126)
	Yeasts (e.g., *Saccharomyces cerevisiae*)	High protein, antioxidants, beta-glucans, ergosterol, reduces methane emissions in ruminants, B-vitamins	Beta-glucans, ergosterol, mannan-oligosaccharides, antioxidants, B-vitamins (B1, B2, B6, B12)	Strain-specific responses, high production cost, Risk of digestive upset if used in excess	Fermentation	(127–129)
Cereal grain processing byproducts	Brewers’ spent grains	Circular feed use	B-vitamins, polyunsaturated fatty acids, phenolic acids, antioxidants	Mycotoxins		(130–132)
	Maize milling byproducts	Circular feed use		Mycotoxins, variable quality		(133)
	Distillers’ dried grains	Circular feed use		Mycotoxins, variable quality		(134–136)

### 6.1 Local production and applicability of climate-smart livestock feeds in semi-arid Southern Africa

#### 6.1.1 Small cereal grains

In the advent of climate change, the traditional small cereal grains may become dietary energy options, despite their previous displacement as staple food crops by improved, maize hybrids. Of the small grains, sorghum (*Sorghum bicolor*) and Pearl millet (*Pennisetum glaucum*) seem to be the most suitable candidates. They are tolerant to heat, drought and low soil fertility, and yield reasonably well under such adverse conditions. Apart from the organic and mineral nutrients, the small grains contain many functional nutrients. Sorghum contains the flavonoids luteolin, kaempferol, quercetin, catechin and the phenolic acids such as ferulic acid, caffeic acid, vanillic acid, p-coumaric acid (91, 92), compounds which help reduce oxidative stress in heat-stressed livestock (78). Pearl millet is rich in the flavonoids tricin and acacetin, as well as phenolic acids such as vanillic and salicylic acids (93).

#### 6.1.2 Grain legumes

In the context of CSLN, two native grain legumes seem to be the most eligible alternative dietary plant protein sources to complement the small cereal grains for livestock feeding in semi-arid Southern Africa. The Cowpea (*Vigna Unguiculata*) is widely cultivated, highly climate and edaphically adaptable, rich in protein, starch, minerals, and the B-group vitamins (94–96). The Marama bean (*Tylosema esculentum*) is a wild, and widely endemic in the region, is protein-rich, drought-resistant, but largely neglected perennial legume (97). In addition to the high protein content, the marama bean is rich in phytochemicals such as phenolic acids, phytosterols, flavonoids, behenic acid and griffonilide, with carbohydrate content in the tubers (97). However, typical of the genus among other undesirable attributes, these leguminous feedstuffs contain high levels of trypsin inhibitors and other toxic antinutrients, which necessitate processing to optimize their nutritional benefits (94).

#### 6.1.3 Roots and tubers

There are a range of climate-resilient indigenous root/tuber crops, many of which are excluded due to the feed-food competition. In this regard, Cassava (*Manihot esculenta*) is an outstanding climate-smart alternative to maize for livestock feeding in semi-arid Southern Africa. Yet to find a firm footing in the region’s agriculture and the food chains, Cassava is drought and heat-tolerant and grows well in poor soils (98). Compared to maize grain, cassava has a higher root biomass and yields more starch at a lower input cost (99, 100). However, along with the leaves, the root periderm contains cyanogenic glycosides, particularly linamarin and lotaustralin, which remain toxic if not properly processed (99).

#### 6.1.4 Browse trees and byproducts

Ruminants in Southern African rangelands browse on many leguminous tree species such as, among others, *Piliostigma thonningii*, *Dichrostachys cinerea*, *Colophospermum mopane*, and *Vachellia karroo*, from which they consume the high-protein pods, twigs, and leaves, mostly during the dry season. These components can alternatively be harvested and processed into bush meal, and similarly for dry-season or drought feeding (101, 102). Bush meal also contains a range of bioactive compounds, including phenolics and flavonoids, with high tannin levels that inhibit protein digestibility. The tannins can be neutralized by supplementary polyethylene glycol (PEG) or can be reduced through soaking and ensiling (103, 104). With proper treatment, bush meal can be a sustainable, climate-smart feed.

A wild, non-legume fabaceous tree feed resource which is abundant in the ecosystem is the Marula (*Sclerocarya birrea* subsp. *Caffra*). Endemic to much of sub-Saharan Africa, the Marula tree produces fruits which are rich in vitamins, amino acids, carbohydrates, organic acids, and polyphenols (105). Livestock consume the fresh fruit’s pulp or its processed byproducts from traditional brewing, such as ensiled or dried pulp. The Marula fruit has a high sugar content which provides dietary energy. The dried pulp preserves most of the essential nutrients, while fermentation enhances the digestibility and introduces beneficial probiotics (106). Climate models suggest increased Marula abundance, which reinforces its potential role as a significant climate-smart feed (107).

#### 6.1.5 Oilseed cakes from climate-resilient plant species

Oil extraction byproducts from a range of wild or cultivated climate-resilient plant species which are common in semi-arid regions, where they have attracted attention as alternative protein and energy options for livestock feeding. Oil cake from the *Macadamia integrifolia* nut contains as much as 19.5% crude protein and is a cost-effective source of dietary energy (108, 109). However, its high fiber content (up to 25%) limits its inclusion in monogastric livestock diets to avoid depressed feed intake and nutrient digestibility (110).

The pharmaceutical oil cake from the castor bean *(Ricinus communis)* is rich in protein and energy, but contains toxins, primarily ricin. Ricin and its poisonous derivatives can be destroyed by moist heat treatment or low pH fermentation (111). The biofuel byproduct from *Jatropha* (*Jatropha curcas*) beans is high in protein and energy but contains toxic phorbol esters. The phorbol esters can be detoxified by heat treatment or fermentation (112).

#### 6.1.6 Forage crops

Two species stand out as potential climate-smart forage resources in the region. One of these is Napier grass (*Pennisetum purpureum*), a high-yielding, drought-tolerant forage crop suitable for semi-arid regions (113). The other one is the Prickly pear (*Opuntia ficus-indica*), which, despite classification as an invasive plant, plays a significant role in supporting rural livelihoods (114). Endemic to arid regions, the Prickly pear is increasingly cultivated for its fruit or forage. The water-rich leaves (cladodes) are the primary livestock forage, which along with the byproducts, namely waste fruit and seed oil extraction cake can be used as feed for livestock (115, 116). Prickly pear feed products are rich in energy, protein, antioxidant flavonoids and phenolic acids, and betalain pigments (betacyanins and betaxanthins) that express antioxidant and anti-inflammatory properties (115, 117). However, the products contain tannins and phytate, which may require processing (118, 119).

#### 6.1.7 Insect feed

The use of alternative, comparatively inferior protein sources such as native legumes for livestock feeding could undermine the formulation of precision diets, and increase the need for expensive supplementary animal protein, such as fishmeal. However, fishmeal is also threatened by climate change and overfishing. Insect feed, particularly the Black Soldier Fly larvae (*Hermetia illucens*), is emerging as a viable alternative (120–122). Black Soldier Fly larvae contain high levels (40–44%) of crude protein, with advantage of efficient, eco-friendly production (120, 123).

#### 6.1.8 Microbial feedstuffs

Phototrophic algae (124–126) and yeasts (127–129) are potential climate-smart feed resources. Subject to the cost, microbial feedstuffs carry the advantage of efficient production of protein in controlled environments, with minimal land and water input, and low environmental impact. In addition to protein, microalgae are rich in carotenoids and polyphenols, which neutralize ROS and mitigate oxidative damage (124, 125). Yeasts produce antioxidants and beta-glucans and ergosterol, which support immune functions and reduce oxidative cell damage (128).

#### 6.1.9 Cereal grain processing by-products

Brewers’ spent grains contain 20–30% crude protein and are rich in B-vitamins such as thiamine and riboflavin (130). They also contain high levels of phenolic acids (130, 131). However, they may be contaminated with mycotoxins (132). Maize milling byproducts (bran, germ meal, gluten feed or meal, hominy chop) contain variable (8–23%) crude protein, are noted for their phenolic compounds such as ferulic acid, which offers antioxidant benefits (133). However, these byproducts may also contain mycotoxins, necessitating careful management to ensure feed quality. Distillers’ dried grains are a byproduct of ethanol production with a high (25–35%) crude protein content and are rich in diversely bioactive compounds (134–136).

#### 6.1.10 Circular feed systems

Similar to circular food systems (137), circular feed systems are more sustainable and reduce the environmental footprint. Examples of the climate-smart feedstuffs in such circular systems include the oilseed cakes, cereal grain processing byproducts and insect feeds efficiency (138).

Circular feed systems emphasize recycling and reusing locally accessible feedstuffs to cut waste and boost system resilience for sustainable feeding of livestock. This strategy fits into CSLN, especially in semi-arid areas where resource limitations and feed scarcity are most intense (139). For example, brewers’ spent grains, oilseed cakes, and fruit pulp are agricultural and agro-industrial by-products that can be recycled into nutritionally balanced livestock meals. While technologies such as composting organic waste or raising insects such as Black Soldier Fly larvae turn waste streams into high-quality protein feeds, dual-purpose crops such as maise and sorghum supply grain for human use and leftovers for livestock feeding (140). Furthermore, livestock dung can improve soil quality, promoting the development of fodder crops and maintaining a closed-loop nutrient cycling system.

Crop residues, by-products, and organic waste are examples of locally accessible resources used as major inputs in this system. To increase feed value and reduce spoilage, these resources are processed using technologies such as fermentation, urea treatment, and silage production. While feedback loops ensure that animal waste, including manure, is returned to the land to increase forage production and promote sustainable agriculture, the outputs are nutrient-dense, inexpensive feeds that satisfy cattle’s energy and protein needs.

## 7 Feed additives and supplements

Where the novel diets lack adequate biofunctional compounds to achieve the climate-smart objectives, a range of synthetic or microbial or plant-derived products can be used. There is a range of feed additives and supplements that enhance animal well-being and performance. These can be natural plant extracts, or synthetics (141). Candidates for adoption in CSLN are described in Table 5.

**Table 5:** Climate-smart feed additives and supplements.

Additive/supplement	Climate-smart attributes	Bioactive compounds	Limitations	References
Phototrophic algae	Sustainable protein source, rich in antioxidants (carotenoids, polyphenols) that neutralize reactive oxygen species (ROS) and mitigate oxidative damage. Enhances livestock thermal resilience and reduces environmental impact	Carotenoids, polyphenols, EPA, DHA	High production costs: scaling challenges for large-scale livestock use	(124–126)
Yeast derivatives	Source of antioxidants, beta-glucans, and ergosterol, supports immune function, reduces oxidative cell damage, and contributes to better stress resilience	Beta-glucans, ergosterol, antioxidants	Strain-specific responses; variable bioavailability	(128, 129)
Probiotics	Modulate gut microbiota, improve digestion and immunity, enhance stress resistance and nutrient use	Lactic acid bacteria, Pediococcus, Bacillus	Strain-specific efficacy, affected by storage and environmental factors	(158, 159)
Prebiotics	Promote beneficial gut microbes, enhance gut integrity, improve nutrient absorption	Galactooligosaccharides (GOS), Mannanoligosaccharides	Limited by diet composition and environmental factors	(161, 164)
Exogenous enzymes	Improve nutrient digestibility and absorption, reduce environmental nitrogen and phosphorus excretion	Phytase, xylanase, cellulase	Sensitive to storage and pH variations; requires careful formulation	(241)
Organic acids	Enhance gut health, reduce harmful bacteria, improve feed efficiency	Propionate, acetate, lactate	Reduced efficacy with improper application or dosage	(193)
Phytogenic extracts	Antioxidant, anti-inflammatory, methane suppression, heat stress reduction	Flavonoids, polyphenols, tannins	Effectiveness depends on plant source and dose	(200, 204)
Postbiotics	Enhance gut barrier, modulate immune response, reduce stress effects	SCFAs, polyamines, bacteriocins	Emerging research: effects not fully understood	(181, 185, 242)
Electrolytes	Maintain water and electrolyte balance during heat stress, reduce impact of climate extremes	Sodium, potassium, chloride ions	High inclusion rates can disrupt acid–base balance	(243)

### 7.1 Methane suppressors

The livestock gut fermentation process produces significant greenhouse gasses, particularly methane, which plays a major role in climate change. A range of additives which include probiotics, exogenous enzymes, plant metabolites and fodder trees, organic acids, and other microbes reduce methane emission (142).

### 7.2 Heat stress modifiers

Heat stress modifiers are a variety of tactics used to lessen the negative impacts of high temperatures on the well-being and output of animals. Animals can escape direct sunshine by being given shade and cover, and they can avoid dehydration by having access to clean, cold water (33, 143). An environment can be made more comfortable by using ventilation systems and airflow control to disperse heat and humidity, as well as cooling equipment such as fans and misters (144). Resilience to high temperatures is further increased by behavioral management techniques and genetic selection for heat tolerance features. During times of heat stress, nutritional modifications are essential, such as changing the content of the feed or increasing the amount of electrolyte supplementation (34, 145). Betaine is an amino acid derivative with advantageous biological characteristics that support its use as a useful supplement during heat exposure (146).

Betaine is one example of an additive that has been shown to be effective in decreasing metabolic heat, improving heat dissipation, and increasing nutrient use in order to mitigate heat stress. It functions as a methyl donor, a chemical prebiotic involved in the methyl transfer reaction in cells, and a micronutrient for microbial cells that increases uptake during osmotic stress (146). Moeckel et al. (147) and DiGiacomo et al. (148) reported betaine’s potential to mitigate heat stress by decreasing energy used and therefore metabolic heat production, while also acting to maintain osmotic balance during thermal challenge. Additionally, it stabilizes the intracellular protein structure by increasing hydrogen bonding between aqueous proteins in the folded state, acting similarly to molecular chaperones (149). When oxidative stress is present, betaine has been demonstrated to decrease the mRNA expression of HSP70 (150). However, utilizing an animal model (151), showed that goats supplemented with betaine and exposed to extended heat stress (42°C, 36 ± 2% RH, 6 h per day, for 16 days) generated noticeably lower amounts of HSP60, HSP70, and HSP90 than goats not supplemented with betaine (151). Additionally, through changes in blood chemistry and cellular metabolism, betaine supplementation may help manage heat stress indirectly. The findings of Hall et al. (152) revealed that there was an improvement in the thermotolerance of cattle-fed betaine during the thermal challenge.

The body typically shows a taurine shortage when under stress (153). For this reason, adding taurine to the diet is crucial. Taurine has positive effects on reducing stress, which may lower the amount of reactive oxygen species and shield mitochondria from oxidative damage (153). As an animal’s cell-mediated immune response weakens in summer, glutamine strengthens it (154). Additionally, glutamine promotes the development of intestinal mucosa, shielding the intestine from harm under a variety of stressful circumstances (155). Broilers subjected to cyclic heat stress showed enhanced immunological response and performance attributes when supplemented with 100 mg/kg GABA (156).

### 7.3 Biotic agents

By producing different metabolites to activate the neurological, endocrine, and immunological systems of hosts, the gut microbiota plays a crucial role in maintaining host health (157). By suppressing pathogens, releasing immunomodulatory and bioactive factors and encouraging the growth of beneficial bacteria, probiotics can restore the ecological stability of the gut microbiota. This can also improve the function of the hypothalamic–pituitary–adrenal axis, one of the main stress response systems, and immunity through the microbiota-gut-brain axis or the microbiota-gut-immune axis (158). The use of probiotics, prebiotics, and synbiotics to modify the gut microbiota has emerged as a promising biotherapy approach for the prevention and treatment of a wide range of illnesses, including stress-related conditions (159).

### 7.4 Prebiotics

The gut microbiota is crucial for energy control and the stress response (160). Prebiotics are substances that the host cannot digest, but can be used to ferment and aid in the reproduction and metabolism of intestinal probiotics for the benefit of the host’s health (161). Research has demonstrated that adding dietary GOS supplements to broiler chickens’ jejunum can reduce the disturbance of intestinal integrity by averting changes in TJs and AJs (162). Furthermore, by upregulating occluding mRNA and protein expression, GOS increase intestinal bifidobacteria in rats and is important in preventing disturbance of intestinal integrity (163). Fructooligosaccharide dietary supplements also reduce *E. coli* and *C. perfringens* while increasing the diversity of Lactobacillus in chickens’ gut. Mannanoligosaccharides inhibit the attachment or colonization of harmful bacteria by preventing their binding to mannan receptors on the mucosal surface, most notably *Salmonella typhimurium* (164). Additionally, mannan-oligosaccharides improve intestinal integrity by raising villus height, goblet cell count, lactobacilli and bifidobacteria populations, and lowering the amount of *E. coli* in chicken ceca (165). In Caco-2 and HT-29 cells, HMO treated with *B. longum infantis* enhanced IL-10 expression and transcription of ZO-1, occludin, and junctional adhesion molecule (JAM)-A mRNA (166). *In vivo* studies on hens exposed to heat stress revealed that adding mannan-oligosaccharides and cello-oligosaccharides (COS) to the diet helped to lessen the effects of heat on intestinal morphology and intestinal barrier function (167). By reducing HS-induced increases in pro-inflammatory cytokines and decreases in intraepithelial lymphocytes, IgA-secreting plasma cells, and mucin formation, the probiotic *B. licheniformis* promotes the GIT mucosal immunity in broilers subjected to thermal challenge (168). By boosting mRNA expression, an IgA secretion of the anti-inflammatory cytokine IL-10, *B. subtilis* B10 promotes the development of mucosal immunity in broiler chickens (169). During a 42-day heat stress phase, oral supplementation of *L. acidophilus* and *S. cerevisiae* probiotics with and without selenium supplementation reduced markers of oxidative stress and hepatic inflammation in rats (170).

### 7.5 Probiotics

Probiotics have been described as “live microorganisms that are beneficial to the health of the host at an adequate intake dose” (171). Probiotics have the ability to modify the composition of intestinal microbes and prevent harmful bacteria from colonizing the intestines. They have been shown to have the capacity to aid in the development of a robust intestinal mucosa protective layer, hence boosting immunity and strengthening the intestinal barrier (172).

Lactic acid bacteria (LAB), such as *Lactobacillus bulgaricus*, *Lactobacillus acidophilus*, *Lactobacillus lactis, Lactobacillus salivarius, Lactobacillus plantarum, Streptococcus thermophilus, Enterococcus faecium, E. faecalis*, and *Bifidobacterium* sp., are the bacterial species now utilized in probiotics (173). Probiotics can also include yeast (*Saccharomyces cerevisiae*) and fungus (*Aspergillus oryzae*) (173). Multiple mechanisms are involved in their action, including neutralizing enterotoxins, promoting gut integrity and maturation, improving growth, preventing inflammation, and modulating the immune system, metabolism, and oxidative stability in fresh meat (174). Probiotics have been shown to enhance gut microbial diversity. To be more precise, *Pediococcus pentosaceus* had a greater average SCFA level and *Bacillus sp.* increased body weight (175).

Heat tolerance is causally correlated with the microbial community, which includes the microbiota’s population, composition, and function (176). Heat-stressed mice showed reduced levels of several probiotics, including *L. murinus* and segmented filamentous bacteria (177). It was reported that Bacteroides were greatly decreased, and Akkermansia was dramatically increased in mice when fecal microbiota from heat-stressed pigs was transplanted (178). These findings suggested that a therapeutically beneficial microbiota may have been added to the heat-stressed animals. Probiotics, prebiotics, and synbiotics have been utilized to prevent or lessen the deleterious effects of stress on physiological equilibrium (179). Under hot temperatures, the gut microbiota can be modulated by probiotic or postbiotic supplementation. Supplementing with probiotics (Bospro, Lacto-Sacc) improved physiological state, particularly thermoregulation, in the summertime (23 to 34°C, relative humidity 65 to 89%) (180). Dietary supplementation with *Bacillus subtilis* reduced heat-induced inflammatory reactions via controlling immunity (78).

### 7.6 Postbiotics

Postbiotics are soluble metabolic products or byproducts secreted by living bacteria or released following bacterial lysis. They are widely used because they contain a variety of signaling molecules that may have antioxidant, immunomodulatory, and anti-inflammatory properties (181). Such as probiotics and prebiotics, postbiotics work in a number of ways to have positive benefits. By preventing the growth of harmful bacteria and promoting the growth of good bacteria, they can alter the makeup of the gut microbiota, improve the operation of the gut barrier, have anti-inflammatory and antioxidant qualities, and influence the immune system (182). Various constituents are present in them, including vitamins, bacteriocins, functional proteins, peptides, SCFAs, polyamines, inactivated microbial cells, and other bioactive metabolites (181).

The addition of 0.3% postbiotics, which are made by *Lactobacillus lantarum*, improves the gut microbiota by increasing the populations of Lactobacillus and caecum total bacteria and decreasing those of Enterobacteriaceae, Salmonella and *Escherichia coli* (183). It has been shown that epithelial colorectal adenocarcinoma cells were partially protected against heat-induced damage to their monolayer integrity by pretreatment with galacto-oligosaccharides prior to heat stress exposure (40 to 42°C) for 24 h (184). Furthermore, by increasing gut-beneficial bacteria, primarily butyrate-producing bacteria, oral therapy with fermented *Saccharomyces cerevisiae* prebiotic for fourteen days prior to heat stress exposure mitigates the negative effects of heat stress (185). The postbiotic *Aspergillus oryzae* enhanced energy-use efficiency, water absorption, and intestinal permeability (186).

### 7.7 Other biogenic additives

The GIT taxa distribution is significantly altered by early insults to the microbiota, which have detrimental effects on the host due to the disruption of stable, selective forces that preserve a homeostatic equilibrium (187). With overlapping metabolic capacities, the reticulo-rumen and hindgut contain enormous species-and strain-level variety (188, 189). The ecological characteristics of the microbiota are critical to the stability of the reticulo-rumen and hindgut ecosystems (188).

The functionality and capacity of gut microbiota to use various substrate groups varies (190). Therefore, greater diversity and richness of these microbiota promote stability and allow for more effective utilization of food resources, making them generally advantageous (191). Therefore, the gut microbiota may be changed into a less desirable and functional condition due to the losses in richness and diversity that occur after a high-grain diet and SARA (189).

Organic acids from animal and plant tissues have been incorporated into livestock feed to improve their performance. It contains propionate, acetate, lactate, butyrate, fumaric, tannic, and caprylic acids. These acids are advantageous to birds’ gastrointestinal health and functionality (192). To improve immunity, nutritional digestibility, growth performance, and avoidance of GIT disorders in broiler chickens, organic acids have been added to feeds or water (193).

### 7.8 Phytogenic plant extracts and essential oils

There are different compounds derived from plants that possess thermally beneficial functional compounds including antioxidant and anti-inflammatory properties that help maintain livestock well-being when facing heat stress. In recent times, there has been an increasing curiosity about the application of phytogenic feed additives (PFA) (71, 194–199). Plant polyphenols, which comprise phenolic acids, flavonoids, 1,2-stilbene compounds, and lignins, are polyhydroxy chemicals that are mostly present in plants’ roots, bark, and leaves (200). Plant flavonoids are naturally occurring antioxidants that enhance cellular viability by releasing hydrogen ions and scavenging oxygen-free radicals by their binding to reactive oxygen species (201). Plant polyphenols can boost endogenous antioxidants, including SOD, CAT, and GSH, in addition to decreasing ROS (202, 203). Plant extracts, including flavonoids and polyphenols, are commonly utilized in cattle to improve product quality, boost immunity, reduce heat stress, and increase feed intake (200). Proanthocyanidins, a polyphenol found in bearberries and green tea, can donate electrons or hydrogen atoms and act as an antioxidant (204). Medicinal plants can improve the pathways leading to mitochondrial activity (205), which can reduce the synthesis of oxidative stress and boost synthesis and supply more energy resources (206). Plant extracts primarily use four mechanisms of action to regulate oxidative stress. First, plant extracts’ antioxidant components can limit pro-oxidative activity by giving metals hydrogen atoms (207). It is well known that phenols are potent antioxidants, with some researchers even arguing that their effectiveness surpasses that of vitamins E and C (208). Second, because plant extracts such as flavonoids have more hydroxyl groups in their skeletons, they may be able to deliver more electrons, which could increase their antioxidant ability (209). Third, by lowering oxygen concentrations and quenching oxygen, plant extracts can increase the antioxidant capacity in animal tissues. This prevents the generation of peroxide while activating antioxidant enzymes (210).

Additional mechanisms of action have been investigated to address the antioxidant capacity of plant extracts. These mechanisms include the modification of key proteins’ expression and activity, interactions with particular proteins essential to intracellular signaling cascades, effects on epigenetic mechanisms, and alteration of the gut microbiota (211, 212).

The addition of plant polyphenol extracts from fenugreek, capsicum and green tea enhanced the intake of dry matter, milk, and milk with 4% fat-corrected milk; it also decreased vaginal temperature, enhanced welfare indices, and enhanced the AT with proteins from the acute phase response and Nrf2-oxidative stress response in dairy cows under heat stress (213). Phenolic PFAs appears to improve performance in primiparous sows and lessen oxidative damage brought on by heat stress (214). Plant flavones, which originate from the phenylpropane metabolic pathway and are secondary metabolites of plants, have been shown to alleviate hypertrophic symptoms in dairy cows (215). Because quercetin has hydroxyl groups and a B-ring twisting angle, it is a flavonoid with a high capacity for antioxidants (216).

### 7.9 Exogenous enzymes

Proteases, phytases and non-starch polysaccharide degrading enzyme (NSPases) improve digestion, to enhance nutrient utilization in livestock exposed to heat stress. Animal diets frequently include supplementation of enzymes, and the physiological effects of these substances are well established. In order to improve nutrient digestion and support livestock growth, feed enzymes have been incorporated into diets on a large scale. It has been shown that the best way for the livestock industry to lower phosphate excretion in animal waste is to incorporate microbial phytase into animal feeds. Additionally, it increases the amino acids availability (217). It has been demonstrated that adding proteases, phytase, and xylanase to the diets of broiler chickens and pigs increases their nutritional value by enhancing nutrient digestibility and growth (217). Furthermore, by lowering the oxidative stress response and possibly affecting the makeup of the mucosal microbiota in the small intestine, these enzymes have shown a functional advantage (217). Research is currently ongoing to determine the exact processes underlying their activities.

Catalysts such as protease, xylanase, and phytase aid in the digestion of proteins, *β*-1,4-xylan linkages, and phytic acid. They may also have benefits for the digestive health and microbiota of chickens and pigs (217). Based on their intended use, commercial enzymes fall into three primary categories: Phytase breaks down fiber into smaller components by targeting phytate molecules, which are generated from phosphorus (218). Cellulases and beta-glucanases, on the other hand, target cellulose polysaccharides and NSPs, respectively. Proteases, on the other hand, work on proteins to improve digestion. In conclusion, alpha-amylase enzymes function as starch and enhance nutrient digestion (219). Depending on specific needs, an animal’s diet may contain a single enzyme or an enzyme cocktail (220). For example, regular digestive tract enzymes can also be used in conjunction with the traditional use of xylanase, glucanase, phytase, and, more recently, multi-carbohydrase preparations (217).

In addition to advantages in the lipid and oxidative profile of meat, a blend of exogenous enzymes (amylase, protease, cellulase, xylanase, and beta glucanase) in the individual and combination form in the feedlot steers diet positively altered nutritional indicators (221). Dairy cows and beef cattle operate more productively when given exogenous fibrolytic enzymes; nevertheless, the right combination of cellulases and xylanases relies on the content of the feed in ruminant diets (222). It is believed that feeding yeast cultures (YC; *Saccharomyces cerevisiae*) and fibrolytic enzymes (cellulases and hemicellulases) made by bacteria and fungi will improve fiber digestion, raise post rumen nutrient flow, and stabilize rumen pH (223). This could be beneficial to cows during heat stress. When xylanase is added to a diet of wheat co-products from flour milling that include high levels of arabinoxylan and NSP, it has been shown to increase energy digestibility in pigs (224). Studies on adding xylanase to broiler chicks have continuously shown benefits, including decreased digesta viscosity and increased nutritional digestibility (225). Energy use has been reported to be enhanced by the phytase enzymes obtained from *Aspergillus niger*, *Peniophoralyci*i, *Schizosaccharomyces pombe*, and *Escherichia coli* (226).

## 8 On-farm feed production

By utilizing the socioeconomic and agro-ecological conditions of the region, semi-arid Southern Africa offers exceptional prospects for the domestic production of climate-smart animal feeds. Among the main feed sources are:

- Crop residues: There are abundant drought-tolerant crop residues in the area, including sorghum, millet, and cowpea, which can be turned into inexpensive animal feed. Their nutritional value can be increased by processing techniques as chopping, urea treatment, or ensiling.
- Forage crops: Because of their high feed quality and resistance to drought, species such as *Leucaena leucocephala*, Stylosanthes spp., and *Cenchrus ciliaris* (buffel grass) are suited for cultivation.
- Agro-industrial by-products: These excellent feed materials, which are in line with the circular economy principles, include brewer’s spent grains, sunflower meal, cottonseed cake, molasses, among other by-products from nearby agricultural sectors.
- Insects and algae: Because of their low resource requirements and capacity to adapt to local conditions, emerging feed sources such as spirulina and larvae of Black Soldier Flies hold promise for scaled production.

The capacity for various farming systems to incorporate different feed interventions varies:

- Smallholder mixed crop-livestock systems: Utilizing crop wastes and forage crops can help these systems, which are prevalent in semi-arid areas. Simple technologies such as hay baling and silage-making can be used by small-scale farmers to preserve food for dry seasons.
- Commercial livestock operations: To increase feed efficiency and lower input costs, larger-scale commercial systems can use hydroponic fodder systems, high-protein concentrates, or processed by-products.
- Pastoral systems: To reduce overgrazing and soil damage, pastoralists in arid regions can use energy-rich feed blocks or supplements to keep livestock alive during times when grass is sparse.
- Agro-pastoral systems: By combining crops that may be used for both food and feed (such as sorghum and maize), these systems can optimize resources.

## 9 Animal genetics and climate-smart livestock nutrition

Climate change threatens livestock productivity through heat extremes which overwhelm artificial climate controls and disrupt animal homeostasis. Heat stress alters the expression of genes which are involved in the control of metabolism and immune responses, which may compromise animal performance (227). As heatwaves increase and intensify, the genetic gains achieved in livestock are therefore at risk. Epigenetics, nutrigenomics and nutrigenetics present different solutions which target the genes for stress tolerance and productivity.

Epigenetics describes heritable changes in gene expression which occur without altering the DNA sequence, which are triggered by environmental factors such as temperature and nutrition. It involves molecular modifications such as DNA methylation and histone protein changes which alter how genes are expressed (68). By identifying specific epigenetic markers, breeders can select animals which are most equipped to cope with heat stress (228). Nutritional epigenomics targets these epigenetic mechanisms through the diet. For example, choline, folate, and betaine can act as methyl donors in epigenetic medications which alter gene expression to mitigate the effects of heat stress (228, 229).

Nutrigenomics is about how dietary nutrients influence the expression of genes which control stress tolerance, metabolism, and productivity. For example, antioxidants and amino acids such as methionine are known to regulate genes involved in stress resistance and metabolic efficiency (230). Additionally, dietary components such as selenium and omega-3 fatty acids have been shown to boost the expression of heat shock proteins and antioxidant enzymes. By targeting specific metabolic pathways, nutrigenomics can be used to enhance thermal tolerance while maintaining productivity and feed efficiency (78).

## 10 Policy interventions

Investments in infrastructure for feed processing and storage, such as silos and pelletisers, are crucial for a successful integration. Climate-smart feed solutions should be adopted by farmer cooperatives and local production should be encouraged by policy initiatives. In order to increase the ability of smallholder farmers and pastoralists, extension services are essential.

A supportive policy is therefore crucial for the success of CSLN. Climate-smart policies should be sensitive to the large variability in the availability and use of land resources between regions, countries and land management systems and in socio-economic conditions, such as wealth, degree of industrialization, institutions and governance, which affect the capacity to respond to climate change (231). Being integral to broader CSA interventions, CSLN may benefit from climate responses such as crop diversification, yield and nutrient improvements, planted area expansion and intensification, which, in risking GHG emissions, conflict with climate change mitigation (232). Therefore, a complex, livestock nutrition focused, land-energy-water-food-livestock-environment nexus approach remains critical to managing the peculiar synergies and trade-offs associated with CSLN interventions (Table 6).

**Table 6:** A policy framework to support climate-smart livestock nutrition practices.

Policy	Action	Deliverables	References
Financial incentives	Provide insurance and financial support to farmers adopting climate-smart feeding practices.	Adoption of climate-smart feeding practices	(1, 10)
Research funding	Invest in research and development for climate-smart feeding technologies.	Innovations and continuous improvement in CSLN	(7, 8)
Regulations on GHG emissions	Develop and implement guidelines and rules to reduce GHG emissions from livestock production systems.	Climate-smart livestock practices to meet legal emission targets.	(1, 232)
Awareness campaigns	Conduct educational programs to inform farmers about the benefits of adopting climate-smart livestock nutrition.	Knowledge and wide-scale behavior change.	(10, 13)
Market incentives	Establish premium markets for sustainably produced livestock products.	Develop climate-smart value chains and economic benefits derived from adopting sustainable practices.	(10, 25)

## 11 Conclusion and recommendations

Southern Africa’s livestock production systems are increasingly challenged by adverse climate change which exacerbates feed scarcity, reduces feed quality, and exposes livestock to thermal stress. The concept of CSLN is a potential solution, whose objectives include adapting to declining feed availability, enhancing livestock resilience to heat stress, and minimizing the environmental footprint of livestock production. The concept of CSLN advocates for alternative, climate-resilient feed sources. To enhance livestock resilience to heat stress, CSLN emphasizes dietary strategies that incorporate natural antioxidants, electrolytes, and polyphenols, which reduce oxidative stress and improve thermoregulation in animals, minimizing the need for synthetic additives, which are an option. For environmental sustainability, precision feeding and the use of circular feed systems are recommended to reduce greenhouse gas emissions and optimize the use of land, water, and energy resources.

Sustainability and feed-food competition must be carefully considered when adopting climate-smart alternative feedstuffs. A workable alternative is provided by dual-purpose crops and tree shrubs, which act as ecological enhancers and feed resources. Their incorporation into agricultural systems could guarantee feed availability while reducing land degradation and deforestation. To improve their production and use at scale, further research is necessary, backed by investments and regulations that serve larger objectives for environmental preservation and food security.

Analyses of the scope for CSLN supported the following key recommendations for its success;

- Developing climate-resilient feed systems: Investment in research and development of a broad range of alternative, sustainable feed sources that are locally adapted to Southern Africa’s semi-arid conditions is critical. This includes insect meal, climate resilient, cultivated and wild forage and food crops, agro and other by-product feedtuffs.
- Improving livestock resilience to thermal stress: Feed formulations which utilize different biotics and phytogenic additives and incorporate synthetic bioactive compounds such as antioxidants and electrolytes that enhance the animals’ ability to cope with heat stress. These strategies should be complemented by selective feed plant and animal breeding for heat tolerance.
- Promoting environmental sustainability: Policies which encourage circular feed systems that reduce resource wastage, lower GHG emissions, and make efficient use of land, water and energy resources through agro-ecological practices.
- Policy and financial incentives: Governments and development organizations to provide financial incentives and insurance schemes to encourage farmers to adopt climate-smart feeding practices and promote the development of climate-smart feed and animal product supply chains.
- Research funding: Research directed toward climate-smart technologies to ensure continuous improvement.
